# Propofol/Fentanyl/Rocuronium or Sevoflurane Inhalational Induction for Intubation?

**DOI:** 10.7759/cureus.19510

**Published:** 2021-11-12

**Authors:** Giakoumis Mitos, Giannoula Thoma, Georgia Tsaousi

**Affiliations:** 1 Anesthesiology, G. Papanikolaou General Hospital, Thessaloniki, GRC; 2 Intensive Care Unit, St. Paul General Hospital, Thessaloniki, GRC; 3 Clinic of Anesthesiology and Intensive Care, AHEPA University Hospital, Faculty of Medicine, School of Health Sciences, Aristotle University of Thessaloniki, Thessaloniki, GRC

**Keywords:** sevoflurane vital capacity induction, sevoflurane vs propofol, sevoflurane apnoea, sevoflurane induction, sevoflurane intubation

## Abstract

Introduction

Anesthesia induction and airway instrumentation are critical parts of anesthesia administration. Intravenous induction is time convenient but necessitates immediate commencement of ventilation. Inhalational sevoflurane induction takes longer but preserves spontaneous respiration. The primary aim of this study is to evaluate the intubation quality features achieved by sevoflurane as the sole induction agent compared with the standard intravenous induction, involving the use of muscle relaxants.

Methods

Sixty patients were randomly allocated into two groups: the Inhalational Vital Capacity Induction With Sevoflurane (IVCIS) group (n = 30) in which patients were intubated after sevoflurane inhalational anesthesia with the vital capacity technique and the Standard Intravenous Induction With Propofol, Fentanyl, and Rocuronium (SIPFR) group (n = 30) after propofol 1.5 mg/kg, fentanyl 2 μg/kg, and rocuronium 0.5 mg/kg administration intravenously. Group IVCIS patients were intubated when bispectral index (BIS) < 60 and end-expiratory sevoflurane ≥ 2 minimum alveolar concentration for > eight minutes. Scoring systems were used to evaluate induction and intubation conditions. The Statistical Package for the Social Sciences (SPSS) software version 25.0 (IBM Corp., Armonk, NY) was used for data analysis.

Results

Intubating and induction conditions were of equal quality in both groups. Sevoflurane induction duration was markedly prolonged. Heart rate was higher in IVCIS group patients throughout the induction, especially during laryngoscopy. Less blood pressure fluctuations were recorded in IVCIS group patients.

Conclusions

Inhalational vital capacity induction with sevoflurane provided acceptable intubating conditions and exhibited a safe hemodynamic profile, albeit the duration was more than 12 minutes.

## Introduction

The combined administration of sedatives, opioids, and neuromuscular blocking agents constitutes a conventional practice of balanced anesthesia induction in both adults and children. Intravenous induction necessitates prompt and immediate airway manipulation maneuvers for the establishment of a patent airway [1]. This may bring the patient and the airway operator in the formidable condition of striving to restore spontaneous breathing or walk through the scenario of unanticipated difficult tracheal intubation in adults.

Inhalational sevoflurane induction has been proposed as a favorable alternative in pediatric or non-compliant adult patients and in cases of difficult airways [2]. Owing to its low blood/gas partition coefficient (0.65), sevoflurane is an excellent choice for rapid and smooth inhalational induction. The main hemodynamic effects of sevoflurane include myocardial contractility depression, vascular resistance, and arterial blood pressure decrease, whilst heart rate is mainly unaffected [3]. Potent inhaled anesthetics seem to depress muscle contractility by yet unknown mechanisms [4]. This property is beneficial since it allows airway instrumentation or specific surgical manipulations, without or with smaller doses of neuromuscular blocking agents. Finally, the main advantage of sevoflurane induction is the preservation of spontaneous ventilation.

Sevoflurane induction has been extensively studied in the pediatric population, albeit only sparse data can be found in the literature that investigates the special features of sevoflurane induction until the establishment of a secure airway in adults. Over and above this, there is no consensus about the appropriate duration or other clinical features of inhalational induction that guarantee safe and effective intubating conditions since most studies investigate the administration of sevoflurane until the induction of anesthesia and not intubation [5].

The present study was primarily conducted to investigate and compare the characteristics of intubating conditions achieved with inhalational or the intravenous induction of anesthesia in surgical patients, with the induction conditions and cardiac effects serving as secondary study outcomes.

## Materials and methods

Study population

Written informed consent was obtained from participants after the nature of the procedure had been explained. This clinical study was performed according to The Code of Ethics of the World Medical Association (Declaration of Helsinki) for experiments involving humans. The study protocol was approved by our Institutional Review Board (AHEPA University Hospital:16th/PN:8929/27.05.2021 ) and registered on clinicaltrials.gov (identifier:NCT04802122).

All consecutive adult patients aged from 18 to 70 years, of both sexes, belonging to the American Society of Anaesthesiologists physical status classification I-III scheduled to undergo elective cholecystectomy under general anesthesia at our institution were eligible for enrolment in this prospective, randomized, and open-label study. Exclusion criteria included obesity defined as body mass index over 34.9 kg/m^2^, presence of abdominal or intracranial hypertension, gastroesophageal reflux, severe hepatic or renal dysfunction, or pregnancy.

Randomization

Based on a computer-generated randomization table (https://sealedenvelope.com), the participants were randomly assigned into one of the two arms with a 1:1 ratio defined as the (a) Inhalational Vital Capacity Induction With Sevoflurane (IVCIS) and (b) Standard Intravenous Induction with Propofol, Fentanyl, and Rocuronium (SIPFR) groups. No use of blocks or stratification was applied to the randomization sequence while assignments specifying only the serial numbers were placed in a sealed envelop.

Preanesthetic assessment

All participants were subjected to a thorough pre-anesthetic assessment the day before the surgery. Upon arrival at the operating theater, routine monitoring involving electrocardiography, pulse oximetry, bispectral index (BIS), and noninvasive blood pressure was instituted for each patient, and baseline recordings were obtained. Furthermore, an arterial cannula under local anesthesia was inserted and cardiac output monitoring (FloTrac™/ Vigileo™ system, Edwards Lifesciences, Irvine, CA) was initiated.

Anesthesia induction

In the IVCIS group, the induction to anesthesia was commenced by sevoflurane 8% (vaporizer setting) and O_2_ 100%, through a primed circuit, using the vital capacity technique. Priming of the circuit was done by connecting a 0.5L breathing bag and a gas sampling line at the Y piece of the breathing circuit while adjusting the sevoflurane vaporizer at 8%, fresh gas flow at 18L/min, and setting intermittent mandatory ventilation according to patients' age and body weight and waiting for end-tidal sevoflurane concentration to reach 2 minimum alveolar concentration (MAC) corrected by the patients' age. After priming, patients were asked to take a vital capacity breath from the residual lung volume, instructed to hold their breath as long as possible, and then perform the same maneuver again until loss of consciousness and return of spontaneous tidal breathing. The end-tidal sevoflurane concentration (SEVOet) was regulated, if needed, by continuous dial adjustments on the sevoflurane vaporizer, to ensure a steady state of two times its MAC. Endotracheal intubation with a proper-sized high-volume, low-pressure, cuffed endotracheal tube was attempted when the SEVOet was two times MAC (4%), for at least eight minutes and the BIS value < 60. In case of apnoea, we applied apnoeic oxygenation in combination with airway release manipulations, waiting for the reoccurrence of spontaneous breathing. The facemask was applied tightly to the patient's face and the adjusting pressure-limiting valve was open to spontaneous mode.

In the SIPFR group, anesthesia was performed by bolus intravenous administration of propofol (1.5 mg/kg) and fentanyl (2 μg/kg) while rocuronium (0.5 mg/kg) was injected to facilitate endotracheal intubation after a three-minute time interval.

In both groups, anesthesia was provided with Leon Plus Anesthesia Workstation (Löwenstein Medical, Bracknell, UK) with a sevoflurane vaporizer (Dräger-Vapor 2000 (Drägerwerk AG & KGaA, Lübeck, DE). The breathing circuit used was double limb adult expandable anesthesia circuits, 1.5L unstressed volume, 90" long, with a 2L breathing bag (Rusch, Teleflex Medical, Westmeath, IE). There was a 1L CO_2_ absorber canister (IntersorbTM Plus Soda Lime, Intersurgical Ltd, Berkshire, UK) attached at the anesthesia workstation.

Anesthesia maintenance

After intubation in both groups, mechanical ventilation was adjusted to a tidal volume of 8 mL/kg, plateau time of 30%, and a ventilatory frequency aiming to keep normocapnia with a fraction of inspired oxygen 40%. Positive end-expiratory pressure was applied to all cases being set at 5 cmH_2_O. Maintenance of anesthesia was ensured with sevoflurane in both groups, targeting a BIS value between 40 and 50. Additional bolus increments of rocuronium were administrated to maintain a post-tetanic count one to five until the cessation of pneumoperitoneum while intraoperative analgesic demands were covered by fentanyl (2 μg/kg) supplemented with remifentanil infusion (0.2-0.5 μg/Kg/min), as appropriate. At the end of the surgery, sugammadex was administered, aiming for a train-of-four ratio (TOFR) > 0.9.

Definitions of primary and secondary variables

The quality of induction in both groups was assessed by the occurrence or not of limb movement (LM), cough (C), salivation (S), laryngospasm (L), and the documented response was graded on a two-point evaluation scale (present = 1, absent = 2). Conventionally, scores ≤ 5 correspond to poor, 6-7 to acceptable, and 8 to excellent induction conditions. During intubation, mandible relaxation (MR), vocal cord position (VCP), airway reaction (AR), and limb movement (LM) were recorded and graded as appropriate (Table 1) [6]. Scores ≤ 7 correspond to poor, 8-11 to acceptable, and 12 to excellent intubating conditions. The laryngoscopic view, as assessed by the Cormack-Lehane classification system, was also documented for every patient.

**Table 1:** Intubating Conditions Grading System Abbreviations: MR, mandible relaxation; VCP, vocal cord position; AR, airway reaction; LM, limb movement

Variable	Grading		
	1	2	3
MR	Rigid	Sufficient	Complete
VCP	Close	Middle position	Adduction
AR	Sustained	Slight	None
LM	Vivid	Moderate	None

Furthermore, heart rate (HR), mean arterial blood pressure (MAP), cardiac output (CO), and stroke volume (SV) recordings were registered at baseline (before the anesthesia induction commencement), before and during the process of intubation, and 10 minutes thereafter.

The primary end-point of the present study was the quality of intubating conditions while induction conditions and concomitant hemodynamic responses in each tested group served as secondary outcomes.

Statistical analysis

A sample size of 27 patients in each group was estimated for a two-sided alpha of 0.05 and power of 80%, assuming a minimum difference in the proportion of patients presenting excellent intubating conditions between groups as being clinically important. Allowing for a 10% drop-out rate, the final study population was set at 60 (30 patients per group). The normality of data was established by the Shapiro-Wilk test. The comparison between groups was performed by the independent T-test or Mann-Whitney test, as appropriate. Repeated-measures analyses of variance or Friedman’s test with Bonferroni adjustment for multiple comparisons were conducted to analyze normally distributed continuous variables over time, or nonparametric data, respectively. The chi-square or Fisher’s exact test was applied to compare the incidence of categorical data variables. Continuous data are presented as mean (standard deviation) or median (range) while nominal factors are reported as counts (proportion, %). For all statistical procedures, a P value of less than 0.05 was considered statistically significant. The Statistical Package for the Social Sciences (SPSS) software version 25.0 (IBM Corp., Armonk, NY) was used for all calculations.

## Results

**Figure 1:**
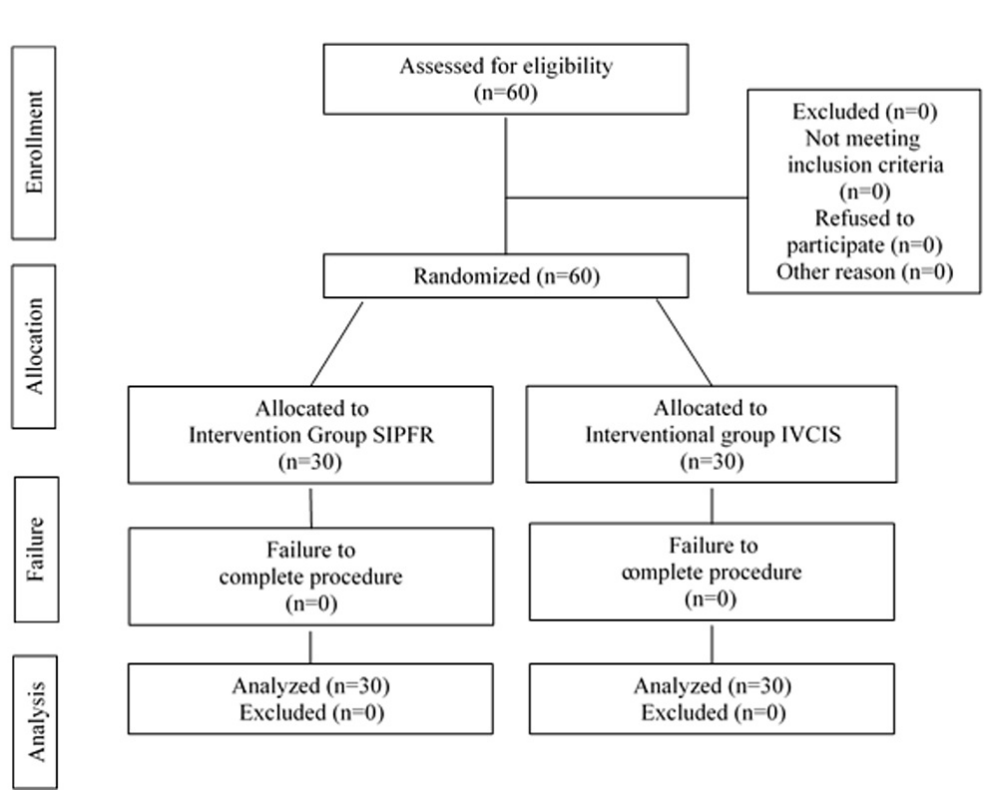
CONSORT Flow Diagram for Patient Allotment

Although a total of 30 patients were recruited in each study arm to compensate for possible dropouts, data from all 60 patients enrolled in the study were available for the final analysis (Figure 1). Demographics, comorbidities, Mallampati score, and intraoperative data were comparable among the two subgroups of the study population (Table 2). No Mallampati score of 4 was documented in any patient.

**Table 2:** Demographic Characteristics and Intra-Operative Data Abbreviations: IVCIS, Inhalational Vital Capacity Induction With Sevoflurane; SIPFR, Standard Intravenous Induction With Propofol, Fentanyl and Rocuronium; NS, not significant. Variables are expressed as means ± SD or counts (percentages)

Variables	Groups		P-value		
	IVCIS (n = 30)	SIPFR (n = 30)			
Age (years)	53.9 ± 12.6	53.1 ± 10.9	t = 0.25	P =0.79	NS
Female gender	18 (60%)	17 (57%)	x^2 ^= 0.06	P =0.79	NS
BMI (kg/m^2^)	26.5 ± 2.5	26.4 ± 3.1	t = 0.09	P =0.92	NS
ASA-PS I-II	23 (77%)	18 (60%)	x^2 ^= 1.31	P =0.52	NS
Mallampati score I-II	28 (93%)	29 (97%)	t = 1.3	P =0.19	NS
Smoking	21 (70%)	11 (37%)	x^2 ^= 0.3	P =0.58	NS
Hypertension	19 (63%)	9 (30%)	x^2 ^= 0.3	P =0.58	NS
Diabetes mellitus	4 (13%)	5 (17%)	x^2 ^= 0.13	P =0.72	NS
Coronary artery disease	3 (10%)	3 (10%)	x^2 ^= 0	P =1	NS
Duration of surgery (min)	85.1 ± 26.8	82.2 ± 34.2	t = 0.36	P =0.71	NS
Duration of anaesthesia (min)	115.5 ± 27.9	112.6 ± 37.9	t = 0.33	P =0.73	NS

The classification of intubation conditions registered in both groups was equally identified as excellent or acceptable (P = 0.79). No case of poor intubation conditions was recorded among the study participants (Table 3).

**Table 3:** Intubation Conditions in Both Groups Abbreviations: IVCIS, Inhalational Vital Capacity Induction With Sevoflurane; SIPFR, Standard Intravenous Induction With Propofol, Fentanyl and Rocuronium; NS, not significant. Data are expressed as absolute numbers and percentages

Intubation conditions	Intubation score	Group IVCIS, n (%)	Group SIPFR, n (%)	P-value
Excellent	12	14 (47%)	15 (50%)	x^2 ^= 0.06, P = 0.79, NS
Acceptable	8-11	16 (53%)	15 (50%)	
Poor	≤7	0	0	

Individual quality features of intubation conditions being achieved either by inhalational or intravenous induction to anesthesia could be regarded as equally satisfactory, considering that each component of the applied algorithm was graded as 3 in the vast majority of cases (Table 4).

**Table 4:** Individual Quality Features of Intubation in Both Groups Abbreviations: IVCIS, Inhalational Vital Capacity Induction With Sevoflurane; SIPFR, Standard Intravenous Induction With Propofol, Fentanyl and Rocuronium; NS, not significant; S, significant. Data are expressed as absolute numbers and percentages

Quality feature / Score	Description	Group IVCIS, n (%)	Group SIPFR, n (%)	P value
Mandible Relaxation				
3	Complete	20(67%)	25(83%)	x^2 ^= 2.22, P = 0.13, NS
2	Sufficient	10(33%)	5(17%)	
1	Rigid	0	0	
Vocal cord position				
3	Open	25(83%)	26(87%)	x^2 ^= 0.13, P = 0.71, NS
2	Middle	5(17%)	4(13%)	
1	Close	0	0	
Airway reaction				
3	None	21(70%)	28(93%)	x^2 ^= 5.45, P = 0.19, S
2	Diaphragm	9(30%)	2(7%)	
1	Maintenance	0	0	
Limb movement				
3	None	23(77%)	26(87%)	x^2 ^= 1, P = 0.31, NS
2	Moderate	7(23%)	4(13%)	
1	Vivid	0	0	

Similarly, no significant difference in the laryngoscopic views, as assessed by the Cormack-Lehane classification system, was registered between the study groups (P = 0.85). In detail, 90% (n: 27) of the patients subjected to either practice were graded as I and II. Furthermore, the duration of intubation was similar between IVCIS and SIPFR groups (6.63 ± 1.43 sec and 6.67 ± 2.35 sec; P = 0.947, respectively).

Overall, similar induction conditions were achieved between the two groups (Table 5).

**Table 5:** Individual Quality Features of Induction in Both Groups Abbreviations: IVCIS, Inhalational Vital Capacity Induction With Sevoflurane; SIPFR, Standard Intravenous Induction With Propofol, Fentanyl and Rocuronium; NS, not significant; S. Data are expressed as absolute numbers and percentages

Quality feature / Score	Description	Group IVCIS n (%)	Group SIPFR n (%)	P-value
Limb movement				
2	No	18 (60%)	20 (67%)	x2 = 0.28, P = 0.59, NS
1	Yes	12 (40%)	10 (33%)	
Cough				
2	No	28 (93%)	27 (90%)	x^2 ^= 0.21, P = 0.64, NS
1	Yes	2 (7%)	3 (10%)	
Salivation				
2	No	28 (93%)	29 (97%)	x2 = 0.35, P = 0,55, NS
1	Yes	2 (7%)	1 (3%)	
Laryngospasm				
2	No	29 (97%)	30 (100%)	P = 1, NS
1	Yes	1 (3%)	0	

Group SIPFR patients had lower MAP values throughout the induction, with the lowest MAP recorded before intubation. In group IVCIS, HR was higher throughout induction, intubation, and 10 minutes after intubation. Patients in group IVCIS underwent a significant increase in HR during intubation, compared to group SIPFR patients (Table 6).

**Table 6:** Hemodynamic Data Abbreviations: IVCIS, Inhalational Vital Capacity Induction With Sevoflurane; SIPFR, Standard Intravenous Induction With Propofol, Fentanyl and Rocuronium; MAP, mean arterial pressure; HR, heart rate; SV, stroke volume; CO, cardiac output. Variables are expressed as mean ± SD a: Significant difference between groups (P=0.006) b: Significant difference between groups (P=0.002) c: Significant difference between groups (P=0.001)

Variable	Baseline		Before intubation		During intubation		10 min after intubation	
	IVCIS	SIPFR	IVCIS	SIPFR	IVCIS	SIPFR	IVCIS	SIPFR
MAP (mmHg)	99.89 ± 13.6	101.50 ± 15.46	86.37 ± 24.3^a^	71.76 ± 13.3^a^	99.22 ± 28	82.10 ± 15.5	78.43 ± 16.9	75.28 ± 11.8
HR (b/min)	80.5 ± 15.40	81.03 ± 15.4	77.93 ± 16.4	75.72 ± 13.6	90.66 ± 24.7^b^	78.3 ± 13.6^b^	82.26 ± 21.3^c^	70.8 ± 13.1^c^
SV (ml)	89.79 ± 30.7	89.20 ± 25.8	70.79 ± 24.6	67.83 ± 28.3	75.15 ± 20.6	74.86 ± 24.7	62.62 ± 21.2	68.03 ± 20.2
CO (L/min)	6.03 ± 2.5	6.01 ± 2.7	4.66 ± 2.2	5.09 ± 2.5	5.64 ± 2.6	5.23 ± 2.5	4.8 ± 2.38	4.66 ± 2.3

There was no patient recorded having BIS <40 prior to intubation or BIS> 60 at any time after the induction of anesthesia.

## Discussion

In this study, we provide evidence that inhalational vital capacity induction with sevoflurane provides acceptable induction and intubating conditions.

The scoring systems used to grade induction and intubation conditions were a four-variable two and three-point system, respectively, which rated conditions as excellent, acceptable, or poor. Such grading systems have been used in the studies of Viby-Mongensen et al., Sivalingam et al., and Thwaites et al. [6-8].

Most patients of the IVCIS group presented diaphragm contraction post-intubation and cuff inflation (slight airway reaction). This was a transient response probably elicited by the stimulus of intubation. All patients who presented this response immediately recommenced spontaneous breathing and there was no need for neuromuscular blocking agent administration. There was no failed intubation in either group. The intubating conditions in our study were better than those described in the study of Muzi et al. and similar to those described in the study of Khangwal et al. [9-10]. The difference is that in these studies patients were manually ventilated after the loss of consciousness.

The most frequent undesirable condition occurring during induction in the IVCIS group was limb movement. The movement of the limbs or torso is related to Guedel anesthesia stage 2. This stage appears to be of prolonged duration during inhalational induction. The study of Hall et al. evinced that inhalational induction with sevoflurane 8% shortens the Guedel stage 2 duration, compared to sevoflurane 3% [11]. The duration of induction in the IVCIS group was much longer (15 ± 2.94 min) than classical induction with intravenous drugs and the use of neuromuscular blocking agents (NMBAs). In the study of Muzi et al., sevoflurane 7% was administered for seven minutes to volunteer adults while bag-mask ventilation was applied, as there was no objective of maintaining spontaneous breathing from the outset [9]. The study of Sigston et al. investigated the appropriate duration of inhalational induction with sevoflurane in children [12]. However, the duration of induction in the pediatric population cannot be compared with that of adults, for reasons of different pharmacokinetics and pharmacodynamics of sevoflurane in the pediatric patient. There are no bibliographical data suggesting clinical indicators of the appropriate time to attempt intubation in adults undergoing inhalational induction. These indicators could be achieving 2MAC sevoflurane (4%SEVOet) or induction duration of 10 minutes as in the study by Katoh et al. [13]. In our study, no patient presented with BIS < 40 throughout the induction period or BIS > 60 during intubation.

The administration of high sevoflurane concentration in the inhaled gas mixture has been associated with a more frequent occurrence of apnoea [14]. Transient apnoea (or breath-holding) during induction was recorded in more than half of patients subjected to the IVCIS technique (63%; n = 19). The average time of onset of apnoea was 46.26 ± 17.04 sec after the initiation of inhalational anesthesia. The duration of apnoea was 38.42 ± 27.99 sec. There was no correlation discovered between the demographic characteristics of the patients and the occurrence of apnoea. In this study, even after the onset of apnoea, no assisted ventilation was applied to the patients. In our study, all patients retained spontaneous breathing after vital capacity induction and there was no need for assisted bag-mask ventilation or use of airway adjuncts to maintain a patent airway. No patient in the IVCIS group who presented with apnoea became hypoxemic or needed assisted ventilation.

The variation in HR during the induction of anesthesia followed the same course in both groups. It is noteworthy that in group IVCIS, HR was higher than in the SIPFR group, throughout the induction of anesthesia. At 10 min after intubation, in the SIPFR group, HR was lower than the HR of IVCIS group patients, nonetheless within normal range limits. These findings agree with the results of other studies on HR variation during sevoflurane administration, showing that by increasing sevoflurane concentration, the heart rate increases [15-16]. The increase in heart rate during laryngoscopy was greater in group IVCIS patients. This may be explained by the fact that no analgesics were administered to blunt the laryngoscopic stimulus. Mean arterial pressure (MAP) was higher in IVCIS group patients throughout induction, during intubation, and 10 min after intubation. The lowest MAP values in the SIPFR group patients were recorded just before intubation. Abolishment of spontaneous breathing, positive pressure ventilation, and the combined effects of anesthetic drugs may explain the reasons why MAP in SIPFR group patients was lower than IVCIS group patients. Blood pressure decreases dose-dependently when administering sevoflurane, due to peripheral vasodilation, decrease in cardiac contractility, disturbance in the function of baroreceptors, and decrease in sympathetic tone [17]. During inhalational induction, there are two clinical problems. First, provoking clinically significant hypotension puts the patient at ischemic risk. Second, despite the administration of sevoflurane, during laryngoscopy, there may be an increase in blood pressure. There is no consensus on the safe limits of blood pressure fluctuation (systolic, diastolic, or mean) during the perioperative period. The risk of blood pressure fluctuation depends mainly on the patient's pathology, the American Society of Anaesthesiologists' physical status, and the nature of the surgery. The risk of complications from hypotension or hypertension depends on the duration of these phenomena and their etiology. During the execution of this study, no excessive decrease or increase in systolic blood pressure (SBP), diastolic blood pressure (DBP), MAP, SV, or CO was observed in the participating population. That should lead to protocol abortion or the need for pharmaceutical corrective interventions.

## Conclusions

Inhalational vital capacity induction with sevoflurane provides acceptable induction and intubation conditions when targeting SEVOet 4% for more than 12 minutes. Breath-holding is a frequent complication of sevoflurane vital capacity induction, but it is transient; it does not cause hypoxemia and does not require manual support of breathing. Regarding hemodynamic parameters, MAP, SV, and CO are maintained within safe parameters during sevoflurane vital capacity induction. The major drawback of sevoflurane induction is its duration, in order to accomplish the perquisites for intubation used in this protocol. Eventually, the technique selected for induction of anesthesia and airway management is dictated by the practitioner's expertise, the patient's clinical status, and the available equipment.
